# Dynamic Bayesian Network Modeling of the Interplay between EGFR and Hedgehog Signaling

**DOI:** 10.1371/journal.pone.0142646

**Published:** 2015-11-16

**Authors:** Holger Fröhlich, Gloria Bahamondez, Frank Götschel, Ulrike Korf

**Affiliations:** 1 Algorithmic Bioinformatics, Institute for Computer Science, c/o Bonn-Aachen International Center for IT (B-IT), University of Bonn, Bonn, Germany; 2 Division of Molecular Genome Analysis, German Cancer Research Center (DKFZ), Heidelberg, Germany; Wayne State University School of Medicine, UNITED STATES

## Abstract

Aberrant activation of sonic Hegdehog (SHH) signaling has been found to disrupt cellular differentiation in many human cancers and to increase proliferation. The SHH pathway is known to cross-talk with EGFR dependent signaling. Recent studies experimentally addressed this interplay in Daoy cells, which are presumable a model system for medulloblastoma, a highly malignant brain tumor that predominately occurs in children. Currently ongoing are several clinical trials for different solid cancers, which are designed to validate the clinical benefits of targeting the SHH in combination with other pathways. This has motivated us to investigate interactions between EGFR and SHH dependent signaling in greater depth. To our knowledge, there is no mathematical model describing the interplay between EGFR and SHH dependent signaling in medulloblastoma so far. Here we come up with a fully probabilistic approach using Dynamic Bayesian Networks (DBNs). To build our model, we made use of literature based knowledge describing SHH and EGFR signaling and integrated gene expression (Illumina) and cellular location dependent time series protein expression data (Reverse Phase Protein Arrays). We validated our model by sub-sampling training data and making Bayesian predictions on the left out test data. Our predictions focusing on key transcription factors and p70S6K, showed a high level of concordance with experimental data. Furthermore, the stability of our model was tested by a parametric bootstrap approach. Stable network features were in agreement with published data. Altogether we believe that our model improved our understanding of the interplay between two highly oncogenic signaling pathways in Daoy cells. This may open new perspectives for the future therapy of Hedghog/EGF-dependent solid tumors.

## Introduction

De-regulation of sonic Hedgehog (SHH) and EGFR dependent signaling pathways have been implicated in about one third of all human cancers [1]. Both pathways are known to interact, but the exact mechanisms are cell and cancer type specific [2]. A recent study characterized this interplay experimentally in Daoy medulloblastoma cells, a presumable model system for medulloblastoma [3]. Medulloblastoma is a highly malignant brain tumor that predominately occurs in children. The authors specifically report overlapping transcriptional downstream effects of EGFR and SHH dependent signaling.

**Fig 1:**
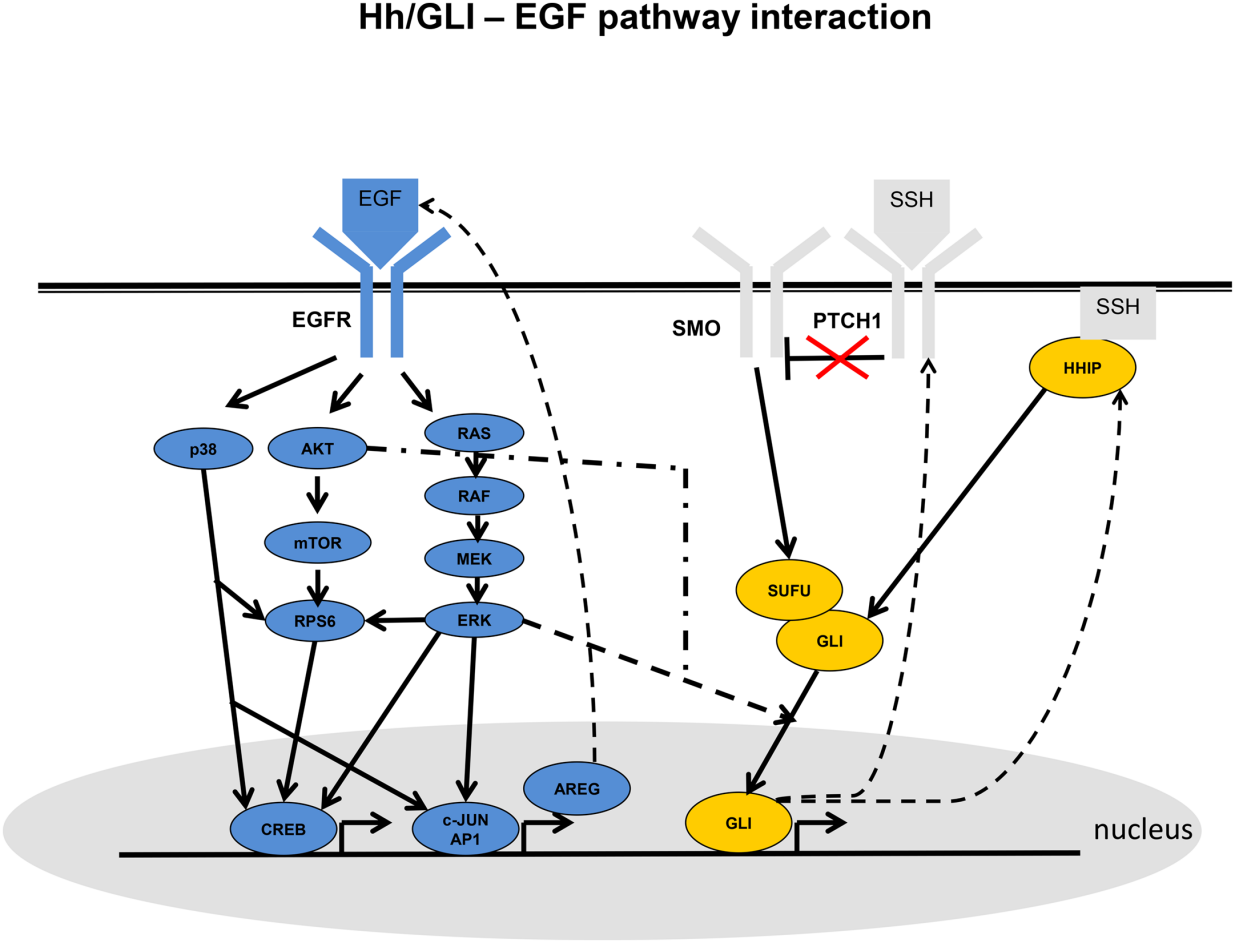
The interaction between EGFR (blue) and sonic hedgehog (SHH, yellow) dependent signaling according to the literature (see references in text): AKT and ERK influence the translocation of GLI proteins into the nucleus, where they function as transcription factors and steer expression of PTCH1 and HHIP (dashed edges). Another transcriptional feedback is given in the EGFR pathway, where c-JUN/AP-1 transcribe AREG, which itself can stimulate the EGF receptor.

According to current knowledge stimulation of EGFR leads to downstream activation of p38, AKT and ERK signaling cascades [4]. These pathways influence protein translation via RPS6 [5–7] as well as gene expression via CREB [8–10]. Furthermore, ERK and p38 yield AP1/c-JUN activation [11], inducing transcription of the EGFR ligand amphiregulin (AREG) [3]. Hence, a feedback loop is established (Fig 1).

According to the common understanding, the cell surface protein PTCH1 blocks the SMO receptor as long as SHH is absent. When SHH binds to PTCH1, this inhibition is released and SMO shows a confirmational change [12]. SMO then allows GLI transcription factors to be phosphorylated, which in the absence of SHH signaling are inhibited by SUFU [13]. It is so far not fully clear, how exactly SHH activates GLI. However, it has been demonstrated that interactions with other pathways, including ERK and AKT, may play a role in human for the response towards SHH [14–16]. Both pathways affect the nuclear localization and activity of GLI1 in a cell type dependent manner. In vertebrates SHH pathway activation can also occur via HHIP in a PTCH1 independent manner [17]. PTCH1 as well as HHIP are transcriptional targets of GLI1 and GLI2, thus forming a feedback loop with potential to further enhance the signaling response towards SHH [18, 19].

Several ongoing clinical trials investigate the therapeutic benefit of targeting the SHH in combination with other pathways in solid tumors, such as PI3K, AKT or mTOR [16]. This motivates to investigate the interplay between the SHH and EGFR dependent pathways in a presumable medulloblastoma model system from a computational point of view. Since signal propagation in biological networks is a time dependent process our focus is on dynamical models. To our knowledge there are no established dynamical models integrating SHH and EGFR dependent pathways in Daoy cells. A major challenge for the development of such models is the dependency of the network structure and dynamical behavior on the investigated cell and cancer type [2]. Moreover, the current understanding of the above described molecular mechanisms is up to a large degree incomplete (for example regarding GLI activation) and purely qualitative. This fact imposes a major difficulty for mathematical model development, e.g. based on ordinary differential equations. On the other hand Boolean Network models [20] are limited by their high abstraction from real cellular processes and have difficulties to deal with stochastic events and noise in real data. Moreover, finding the correct Boolean logic with incomplete knowledge is usually highly challenging [21].

For these reasons we propose a fully probabilistic modeling approach using Dynamic Bayesian Networks (DBNs). DBNs naturally deal with noise in the data. They are not limited to Boolean logic. Furthermore, they allow for scoring and optimizing a given network structure with respect to experimental data. Finally, as demonstrated in this paper, DBNs can be used as predictive models.

In the past static and dynamic Bayesian Networks have been mainly used to learn the topology of molecular networks from a single data source [22–28]. Here we come up with a method to integrate gene and cellular location dependent protein expression data into one DBN model. Furthermore, we demonstrate that DBNs can be used as fully predictive models. Hence, our approach allows us for testing our DBN within a framework that resembles cross-validation in supervised learning.

## Results

### Experimental Data

All experimental data used in this paper have been described previously in Götschel et al. [3]: 13 proteins known to be involved in EGFR and SHH signaling were measured in Daoy cells, which are presumably derived from a medulloblastoma tumor (ATCC: HTB-186). Measurements were performed in the cytoplasm and the nucleus at 14 time points using reverse phase protein microarrays (RPPA) for three different experimental conditions including stimulation with EGF, SHH and EGF plus SHH combined and a series of negative control time points. All measurements were done with 3 biological and 3 technical replicates. In addition, gene expression profiling data for the same time points (Illumina, GEO GSE46045) were obtained in triplicates. Instead of SHH stimulation GLI1 induction was recorded.

**Fig 2:**
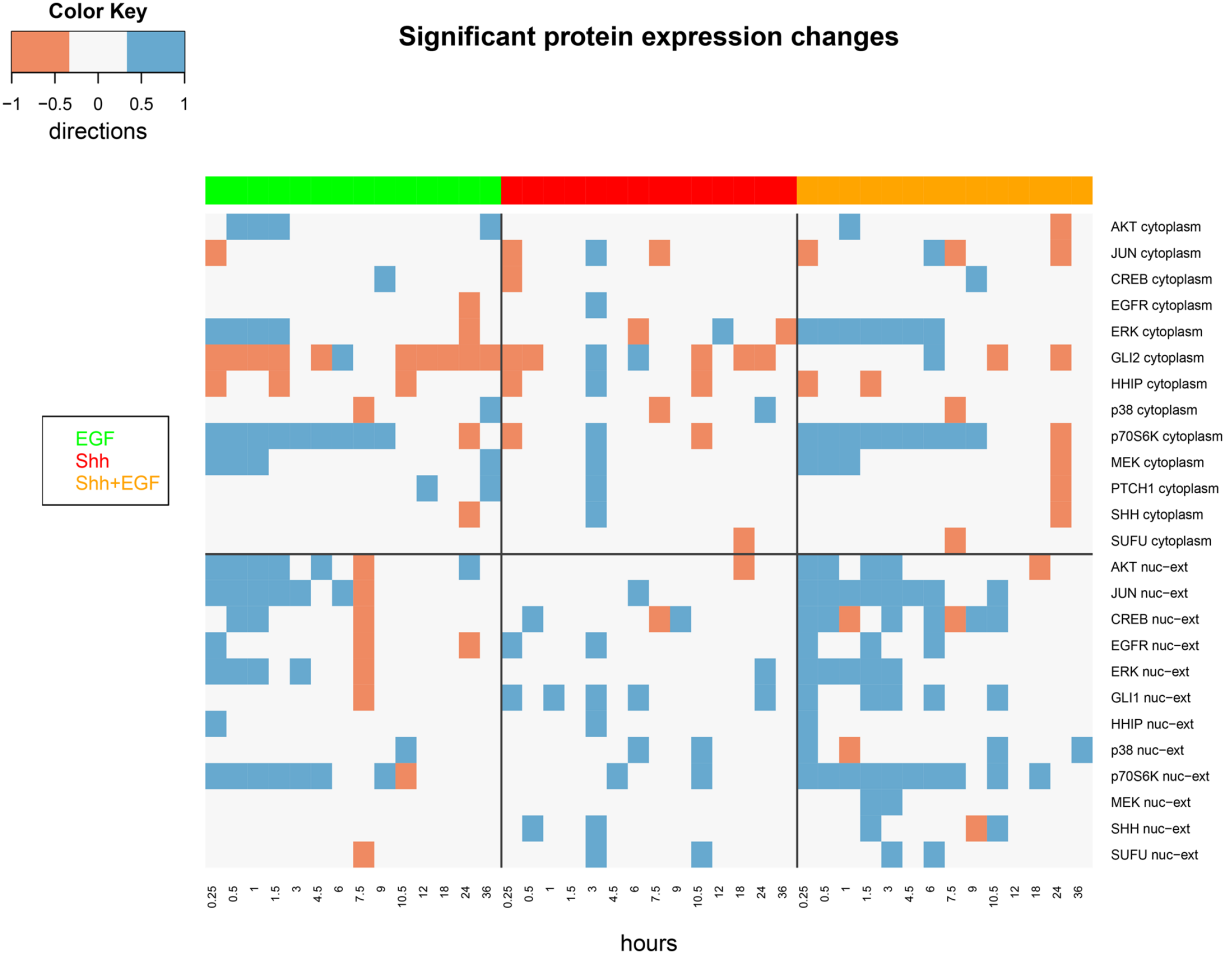
Heatmap showing significant activation (blue) and de-activation (red) of proteins at different time points and stimulation conditions in the nucleus and cytoplasm. Significances are reported at a FDR cutoff of 5%. Proteins without any significant results are not shown.

**Fig 3:**
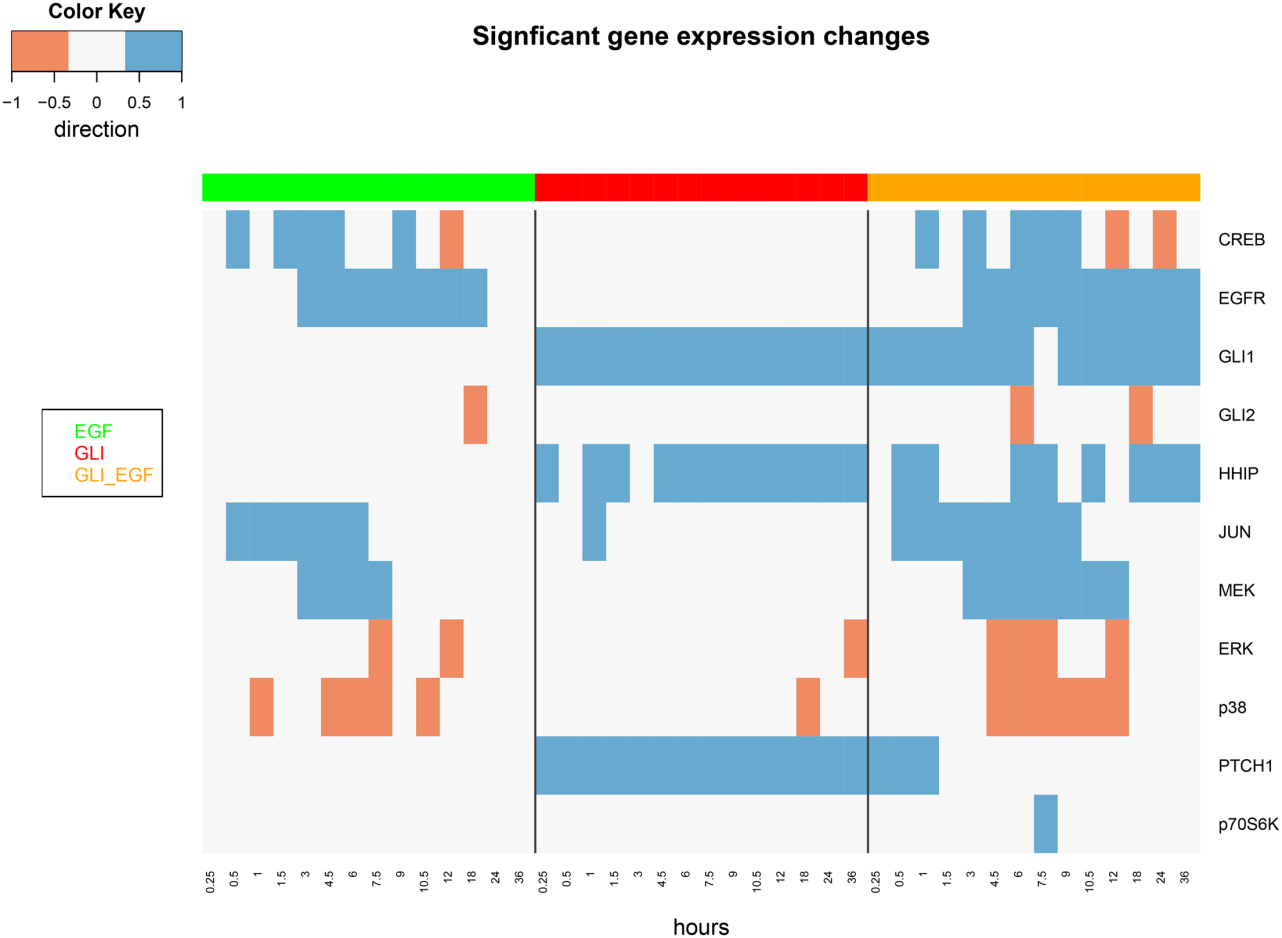
Heatmap showing significant activation (blue) and de-activation (red) of genes at different time points and stimulation conditions. Significances are reported at a FDR cutoff of 5%. Genes without any significant results are not shown.

A statistical analysis of the gene and protein expression data was conducted separately in order to assess whether at a certain time point under a given condition a particular molecule was differentially up-regulated (1), down-regulated (-1) or not significantly changed (0) compared to control (see Section “Analysis of Experimental Data”). The result can be visualized in form of heatmaps (Figs 2 and 3). Differential expression was assessed at false discovery rate (FDR) cutoff of 5%. Specifically on gene expression a relatively clear distinction between EGFR and SHH pathway stimulation was observed. The combined EGF/SHH and EGF/GLI stimulations yielded almost additive effects.

### Dynamic Bayesian Network (DBN) Modeling

In Götschel et al. [3] interactions between SHH and EGFR dependent signaling pathways were merely analyzed from a biological view point, omitting efforts to describe the complex data set as mathematical model. Here we come up with a probabilistic graphical modeling approach using Dynamic Bayesian Networks (DBNs) (Section “Dynamic Bayesian Networks (DBNs)”). A DBN describes conditional statistical dependencies between random variables in a time dependent manner. In our approach random variables reflect the 13 measured proteins. In addition, the biological context of the associated measurements (protein in cytoplasm, protein in nucleus or gene expression) is represented by a variable “context”, which is a parent node of all others. Moreover, the different stimulation conditions are modeled via three extra binary variables “EGF_stim”, “SHH_sim” and “GLI_sim”, which are parents of nodes EGFR (for EGF_stim), PTCH1/HHIP (for SHH_stim) and GLI1 (for GLI_stim). Altogether the DBN model thus comprises 13 + 4 variables. Due to these modeling decisions the experimental context of each measurement can be correctly represented and the data thus integrated. More specifically, we discretize all data into significantly up-regulated (1), significantly down-regulated (-1) and no significant change (0). Statistical significance here refers to a false discovery rate (FDR) cutoff of 5% following the above describe statistical analysis.

The use of intervention nodes (here: EGF_stim, SHH_stim, GLI_stim) to model uncertain, combinatorial perturbations has been previously suggested by Eaton and Murphy [29]. An advantage compared to the ideal intervention scheme [30] is that no assumptions on the actual efficacy of a stimulation are required. Instead, the idea is that a stimulation can alter the conditional probabilities by which a defined target protein is activated or de-activated. Moreover, intervention nodes enable to predict perturbation effects, which are not included into the training data (see below). This would not be possible within the ideal intervention scheme, because there is no explicit representation of perturbations via extra variables.

Having defined the variables in our DBN model the next step is to learn its network topology (or graph structure). For this purpose we use an established Dynamic Programming algorithm [31], which returns a provably optimal network with respect to some score. Here we use the mutual information test (MIT) score [32], which has been suggested to be particularly well suited for biological network structure learning [33, 34]. The MIT score requires the specification of an expected proportion of false positive edges (type I error rate), here 10%.

A major problem with all structure learning approaches is that the true network structure may not be uniquely identifiable from the given data due to the large size of the network space. Hence, integration of prior biological information has been suggested by several authors as a promising strategy [35–38]. Here we employ and compare three different approaches:

1. **strong background knowledge**: For each node only possibly parents are restricted to those described in the literature (Fig 1). Out of these parents the algorithm may choose any subset, based on the given training data.
2. **weak background knowledge**: For each node all other nodes are possible parents, but literature described ones are given a higher probability. Practically this is achieved by rising the type I error rate cutoff for these edges by a specified factor (here two).
3. **no background knowledge**: No background knowledge is used, i.e. structure learning is done completely data driven.

We also learn the parameters, i.e. the conditional probabilities, of the obtained optimal network structure. For this purpose we add a pseudo-count of 1 to observed relative frequencies [39], which is equivalent to a Dirichlet prior.

### Bayesian Predictions agree with Experimental Data

**Fig 4:**
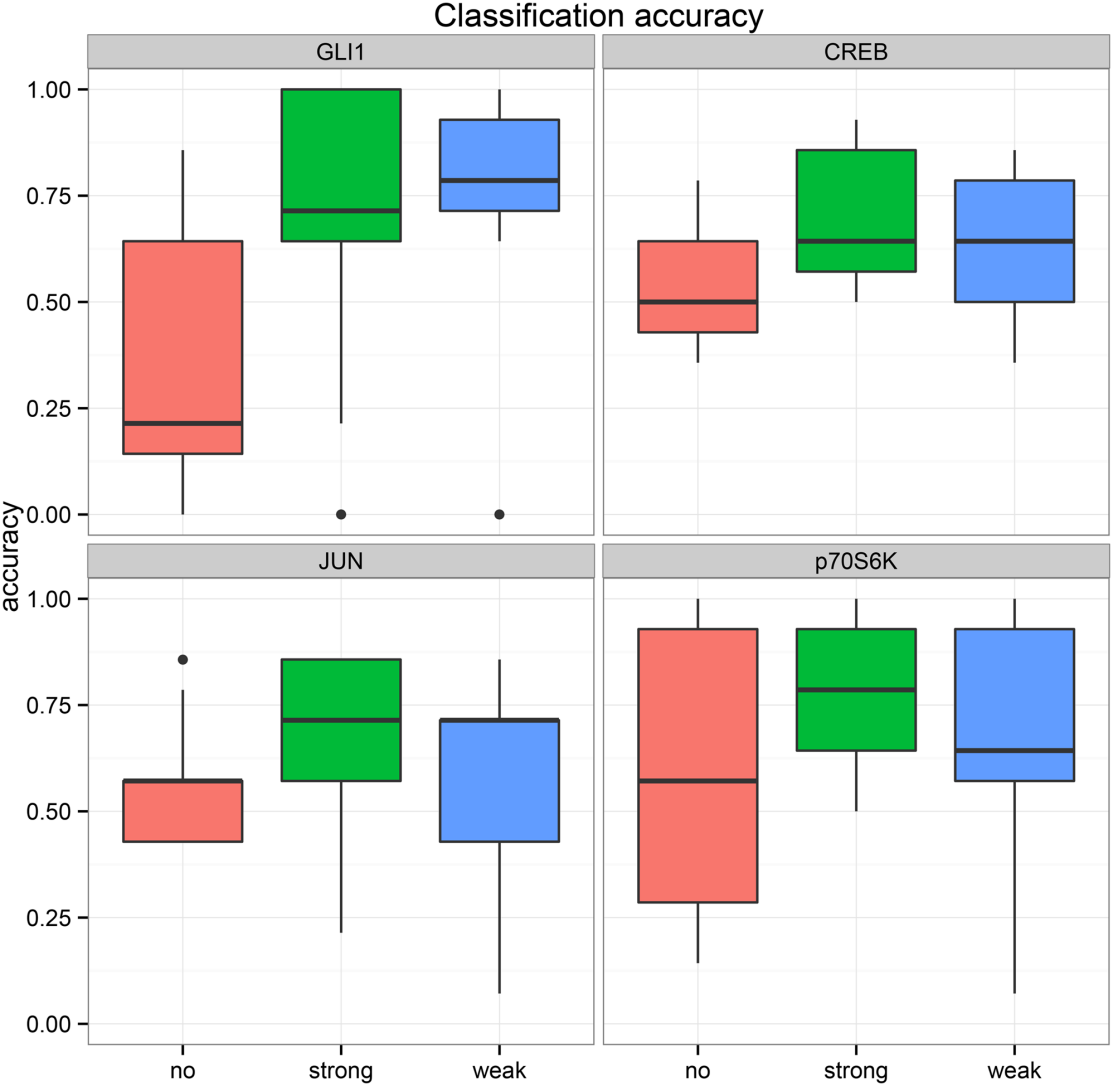
Bayesian predictions for transcription factors CREB, JUN and GLI1 as well as p70s6K on independent time series data. Depicted is the average accuracy for predicting up-regulation, down-regulation or no change using no background knowledge (no), strong background knowledge (strong) or weak background knowledge (weak).

To validate our DBN model based on experimental data we followed the idea that a valid cancer network model should be able to correctly predict the activation state of known key molecules associated to cell proliferation. Therefore, we sequentially left out one of the nine available time series (reflecting different biological context and perturbations) for testing and trained a DBN on the remaining data (using the strategy explained above). Predictions were made for transcription factors GLI1, CREB and JUN as well as p70S6K, which regulates protein synthesis by phosphorylating RPS6. In order to make predictions we implemented a sequential importance sampling algorithm, which allows to estimate posterior probabilities for the activation state for each of these molecules in a fully Bayesian manner (Section “Bayesian Predictions with DBNs”). We then compared for each key molecule and time point the most probable estimated state (i.e. de-activated, activated or unchanged) against the measured one. Fig 4 depicts the accuracies of these predictions for all three model variants. The boxplots show the distribution of accuracies obtained for all 9 tested time series. It can be observed that without background knowledge predictions are significantly worse than with inclusion of information about the network structure. When using background information median accuracies for all molecules are ~75%, which is clearly above chance level. Only small differences are noticed for integrating background knowledge at a weak or strong level. From a conservative point of view this indicates that the model variant “strong background knowledge” is sufficient to explain the data, i.e. it is unlikely that yet unknown interactions exist apart from those described in the literature.

### Stable Network Features agree with the Literature and allow for Model Interpretation

As a next step we focused on the actual network topology of our DBN model variant “strong background knowledge” in more depth. Notice that all edges included in this model variant are supported by the current literature. However, this does not answer the question, how robust and confident individual edges can be identified from the given data. For this purpose we performed a parametric bootstrap. A parametric bootstrap is a sampling based strategy and addresses the question, which parts of the DBN model could have also been learned from other datasets of the same size as the given one (Section “Parametric Bootstrap for DBNs”). While repeatedly (here: 100 times) drawing such datasets one can re-estimate the DBN and count, how often a specific edge is observed despite of differences in the actual training data. Edges observed more often have a stronger support by the data, because they can be learned more stably and robustly.

**Fig 5:**
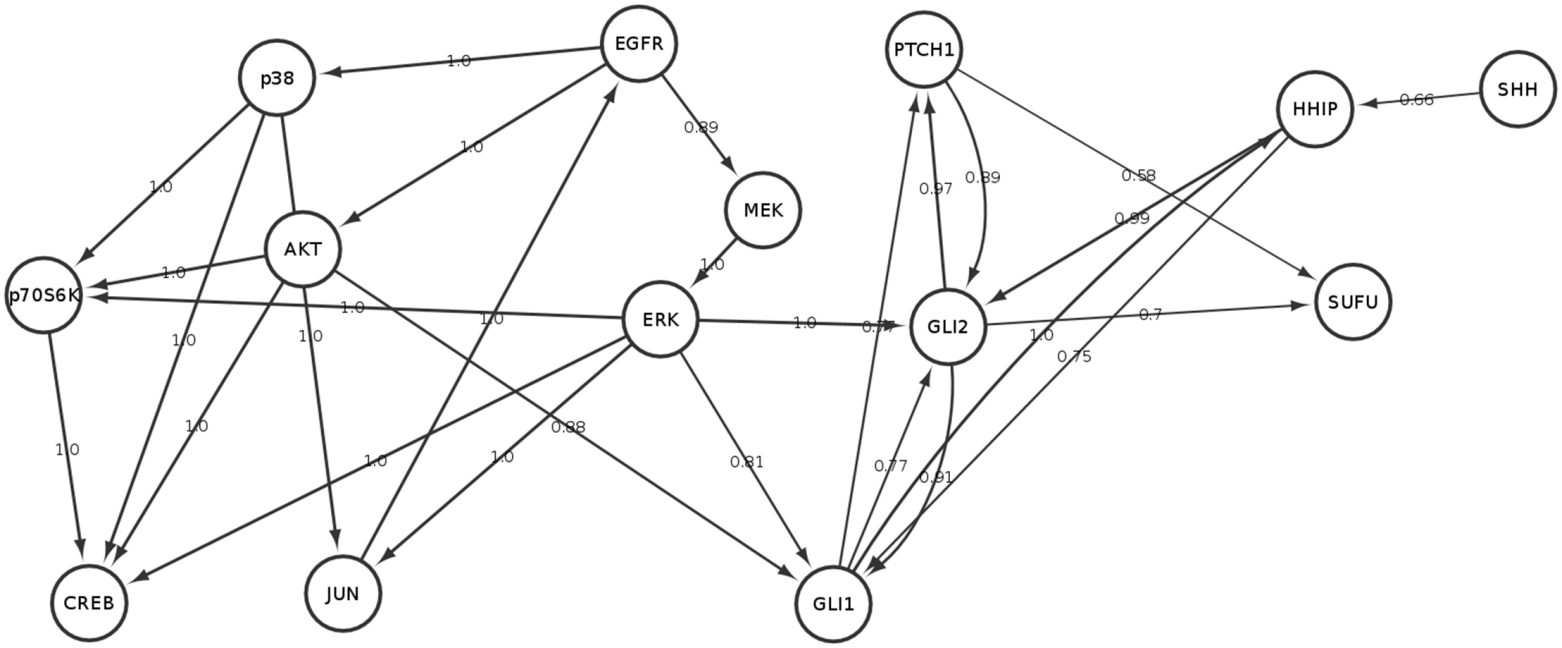
Stable network features learned from data (using strong background knowledge): Edge labels indicate relative parametric bootstrap frequencies. Only edges above a cutoff of 50% are shown and additional nodes “EGF_stim”, “SHH_stim”, “GLI_stim” and “context” have been removed. Moreover, all self-loops have been removed.

We specifically reported edges observed with a frequency higher than 50% (Fig 5). A few interactions fell below this cutoff: We originally included SHH into the parent set of PTCH1, but the bootstrap frequency for the edge *SHH* → *PTCH*1 was only 45%. Hence, in Daoy cells the SHH pathway might predominately be activated via HHIP. Indeed our protein expression data (Fig 2) shows a much stronger dynamical behavior for HHIP than for PTCH1 after SHH stimulation, which supports this hypothesis.

Another example is the edge *PTCH*1 → *GLI*1, which has a bootstrap frequency of 48%, suggesting that SHH signaling in Daoy cells in most cases first activates GLI2 and then GLI1 (because of the highly stable edges *PTCH*1 → *GLI*2 and *GLI*2 → *GLI*1). In agreement we find in the protein expression data that GLI2 is only active in the cytoplasm, while GLI1 is only active in the nucleus.

Looking at stable network features also allows to get insights about the role of SUFU. Our network suggests that SUFU does not directly influence the activation of GLI proteins. However, SUFU protein expression seems to be modulated by GLI2 and takes place in the cytoplasm according to our protein expression data.

Bootstrap frequencies in the EGFR pathway are all close to 1, indicating a high agreement of literature derived knowledge to the existing data. This specifically includes the regulation of CREB, which is a major transcription factor downstream of the EGFR. The edges *ERK* → *GLI*1, *ERK* → *GLI*2 and *AKT* → *GLI*1 reflect the current knowledge on the cross-talk between EGFR and SHH dependent signaling pathways (Fig 1). Once again combining protein expression data with network information indicates that EGF stimulation alone inhibits GLI2 via ERK in the cytoplasm. No further activation of GLI1 on protein level takes place. This in turn indicates that GLI2 down-regulation here compensates the activation of GLI1 via ERK and AKT. On the other hand, activation of GLI1 is the consequence of SHH pathway stimulation without EGF. GLI1 expresses—as reflected by the gene expression data—PTCH1 and HHIP, which constitutes two feedback loops.

In the presence of combined EGFR and SHH pathway stimulation GLI2 activation via HHIP and PTCH1 is compensated by the inhibitory ERK influence, which overall results in a very weak dynamical change of GLI2 protein expression in the cytoplasm. GLI1 activation in the nucleus and on transcriptional level look qualitatively rather similar to the situation when only the SHH pathway is stimulated. The reason could be the activating influence of ERK and AKT. Interestingly, PTCH1 transcriptional activation is shorter under combined EGF / GLI stimulation than under SHH pathway activation alone. This may be explained by the weaker GLI2 influence.

## Discussion

EGFR and SHH signaling pathways interact in a complex way to influence cell proliferation and differentiation. This is of high relevance for many human cancers, including medulloblastoma. Clinical trials in different solid tumors have been started to investigate the therapeutic benefit of combined SHH and EGFR treatment. This motivates to computationally model the interactions between SHH and EGFR dependent pathways in Daoy cells as a presumable model system for medulloblastoma. To our knowledge this has not been done so far.

Here we developed a Dynamic Bayesian Network (DBN) based approach, in which we integrated heterogeneous gene and protein expression data together with prior knowledge from the literature. We systematically tested our DBN on parts of our data that had not been used for training using a Bayesian prediction algorithm. Our approach thus resembles a cross-validation based model assessment, which is commonly used for supervised learning models. Notably, independent test data comprised different biological context and conditions than original training data. Our results indicate that a DBN model starting from current literature knowledge and then being adapted to measured training data is sufficiently able to predict the activities of key molecules associated to cell differentiation and proliferation, thus providing a model validation. In addition, a parametric bootstrap allowed us to get insights into statistically stable network features. All high confidence edges reflect current literature knowledge. Together with our statistical analysis of the experimental data this helped us to interpret the behavior of the modeled biological system.

Altogether we believe that our work improved our understanding of the interactions between two highly relevant signaling pathways in cancer. This may open new perspectives for the future therapy of Hedghog/EGF-dependent tumors.

## Materials and Methods

### Analysis of Experimental Data

#### Protein Expression

RPPA data (S1 File) was obtained from 3 biological and 3 technical replicates for each of the 13 proteins under study. The data was measured under control conditions as well as under EGF, SHH and combined EGF/SHH stimulation in the cytoplasm and the nucleus at 14 time points. After a log transformation of the data we estimated the correlation between technical replicates for each protein via a mixed linear model [40] using the appropriate function implemented in limma [41]. Afterwards the observable mean-variance trend was estimated using data point specific precision weights, taking into account the correlations between technical replicates [42]. These weights were incorporated into a limma analysis to estimate the time, treatment and compartment specific stimulus effect for each protein. For that purpose we defined for each protein a linear model, in which the normalized expression was modeled via two factors “group” and “replicate”, where “group” was created from all possible combinations of treatment, time and compartment, and “replicate” indicated the respective biological replicate. We used a robust empirical Bayes estimate of the variance [43]. The result of the robust limma analysis was a protein specific log fold change together with the corresponding p-value and FDR per treatment, compartment and time point.

RPPAs are an antibody based technique, and in several cases our data contained measurements of the same protein with different antibodies. Some of these antibodies specifically detect phospho proteins. Our above described limma analysis was done on the basis of these antibody specific measurements. However, in our DBN model we used only one variable per protein and in particular did not distinguish between phospho and total protein concentration changes. In order to summarize limma results we first discretized log fold changes into significantly up-regulated (1), significantly down-regulated (-1) and no significant change (0). Statistical significance here refers to a false discovery rate (FDR) cutoff of 5%. Afterwards we considered the sign of averaged discretized values as the overall summary per protein.

#### Gene Expression

Gene expression data (GEO GSE46045) was available in biological triplicates for the same time points as the RPPA data. Instead of SHH stimulation, GLI1 stimulation was measured.

We performed quantile normalization and estimated the mean-variance trend as described above. Only probes with a detection p-value below 1% were considered. A robust limma analysis was conducted and results discretized and summarized per network variable in the same way as described before.

### Dynamic Bayesian Networks (DBNs)

Dynamic Bayesian Networks (DBNs) belong to the families of probabilistic graphical and probabilistic dynamical models [44]. A DBN M can be defined as a pair (Γ,**Θ**) of a directed graph Γ and parameters Θ. Vertices **X** = {*X*_1_,*X*_2_, …, *X*_*n*_} in Γ represent random variables. Edges encode conditional statistical dependences. More specifically each *X*_*i*_(*t*), *i* = 1, 2, …, *n* is conditionally independent of all its non-descendants, given parents *pa*(*X*_*i*_(*t*)). Here *X*_*i*_(*t*) denotes the distribution of *X*_*i*_ at time *t*. The DBN assumes a first order Markov process [22]:

$$\begin{array}{c} P(X(t)|X(0),...,X(T))=P(X(t)|X(t-1)) \end{array}$$

If all nodes are statistically independent at time 0 and for *t* > 0 each *pa*(*X*_*i*_(*t*)) ⊆ **X**(*t* − 1), any cyclic DBN graph can be “unrolled” over time such that all edges point from time slice *t* to time slice *t* + 1. A possible extension of this idea (not used here) is that also within each time slice random variables are allowed to form an acyclic graph [22].

Denoting by **x**(*t*) the joint configuration of *X*_1_(*t*), *X*_2_(*t*), …, *X*_*n*_(*t*) at time *t* the DBN model implies that

$$\begin{array}{c} p\left(x\left(0\right),...,x\left(T\right)|\Theta \right)=p\left(x\left(0\right)|\Theta \right)\prod_{t=1}^{T}\prod_{i=1}^{n}p\left(x_{i}\left(t\right)|pa\left(X_{i}\left(t\right)\right),\Theta_{i}\right) \end{array}$$

DBN learning comprises two fundamental tasks:

- estimation of stationary parameters **Θ** = *P*(**X**(*t*)∣**X**(*t* − 1)) associated to random variables **X** from data
- learning of the graph structure Γ from data

The second task is known to be NP hard, since the number of possible graph structures scales with *O*(2^*n*^2^^) [45]. Moreover, for discrete data with *k* possible values (here: -1, 0, 1) also the first task is NP hard, because for each node *X* there are *k* ^|*pa*(*X*)|^ possible parent configurations. Using appropriate priors (e.g. Dirichlet distributions) is thus highly important (see [46] for an excellent overview).

### Bayesian Predictions with DBNs

We can use DBNs to make predictions on independent test data, given that the graph structure Γ and parameters Θ have been learned from training data. Here we implemented a sequential importance sampling algorithm for this purpose [46] (S2 File). Briefly, the idea is to start with drawing *N* random configurations **x**(0) using learned parameters Θ. For any of these samples **x**_*s*_(0) we compute its likelihood weight ∏_*o* ∈ *O*_ *p*(*o*∣*X*(0) = **x**_*s*_(0)), where *O* is the set of observed variables within the test data. For *t* = 1, …, *T* we then sample parent configurations **x**(*t* − 1) proportional to the likelihood weights. The algorithm now considers these sampled parent configurations to draw from the defined conditional probability distribution for any unobserved node, for which we want to make predictions. Observed nodes are again used to compute likelihood weights, which are then in turn are used in the next iteration of the algorithm to sample suitable parent configurations.

Given that *N* is sufficiently large (here 1000) the algorithm allows to obtain realistic estimates of the probability that an unobserved node *U* at time point *t* has a value of *v*, given the rest of the test data. That means we approximate

$$\begin{array}{c} P\left(U\left(t\right)=v|o\left(1\right),...,o\left(T\right)\right)\approx \frac{\sum_{s=1}^{N}w_{s}\left(t\right)I\left\{u_{s}\left(t\right)=v\right\}}{\sum_{s}w_{s}\left(t\right)} \end{array}$$

were {*w*_*s*_(*t*)} and {*u*_*s*_(*t*)} denote likelihood weights and sampled values of *U*(*t*), respectively. Furthermore, **o**(*t*) denotes observed measurements at time *t*.

### Parametric Bootstrap for DBNs

The parametric bootstrap for Bayesian Networks has already been discussed in [47]. The approach is particularly well applicable to time series data. Briefly, the idea is to start with learning the graph structure Γ and parameters **Θ** from the *complete* dataset *D*. Using Γ and **Θ** we then sample *N* random datasets. For each of these datasets we run a complete DBN structure learning. At the end we count the relative frequency of observed edges in all *N* DBNs. The core question, which a parametric bootstrap addresses via simulation, is the following: Given that the true model (learned from the complete data *D*) was indeed M. Would it be possible to induce M also from other datasets of the same size coming from the same statistical distribution? By answering this question we can determine the level of confidence in features (= edges) of M.

## Supporting Information

S1 File RPPA data. (ZIP)

S2 File R codes. (ZIP)
